# HIV Infection Elicits Differential Transcriptomic Remodeling in CD4+ T Cells with Variable Proliferative Responses to the T Cell Receptor Stimulus

**DOI:** 10.3390/pathogens12040511

**Published:** 2023-03-24

**Authors:** Xinlian Zhang, Savitha Deshmukh, Amey Mukim, Jasen Zhang, Nadejda Beliakova-Bethell

**Affiliations:** 1 Department of Family Medicine and Public Health, University of California San Diego, La Jolla, CA 92093, USA; xizhang@health.ucsd.edu (X.Z.); jasenzhang@g.ucla.edu (J.Z.); 2 VA San Diego Healthcare System and Veterans Medical Research Foundation, San Diego, CA 92161, USA; 3 Department of Medicine, University of California San Diego, La Jolla, CA 92093, USA

**Keywords:** HIV infection, response to T cell receptor stimulus, transcriptomic remodeling, single-cell RNA-Seq, biomarkers of HIV latency

## Abstract

Identification of a cellular biomarker of latent HIV infection will facilitate the latent reservoir detection, quantification, and targeting for elimination. Unfortunately, the latency biomarkers reported in the literature define only a fraction of the entire reservoir. The latent HIV reservoir may be established in dividing cells that subsequently return to quiescence and in resting cells. The strength of the T cell receptor (TCR) signaling at the time of infection affects characteristics of the established reservoir, such as the ability to reactivate with latency reversing agents. To better understand the cellular environments before latency establishment, we characterized transcriptomic remodeling induced by the initial HIV infection in cells with differential proliferative responses to the TCR stimulus. Cell proliferation was monitored using the viable dye carboxyfluorescein diacetate succinimidyl ester. Cells that divided many times, a few times, or remained non-dividing were subjected to single-cell RNA sequencing. A subset of identified transcriptional changes induced by HIV infection was independent of the number of cell divisions; however, responses unique to different cell subsets were also detected. Some of these early gene expression changes were consistent with reported markers of latently infected cells. We pose that the latency biomarkers may depend on the cellular proliferative state at the time of infection.

## 1. Introduction

The main roadblock to eliminating HIV infection is the persistence of the cell reservoir bearing the latent provirus [1]. Identification of the molecular signature, or a “biomarker”, of this latent reservoir will facilitate its detection, quantification, and targeting of these cells for elimination. So far, the identification of universal molecular signatures of cells latently infected with HIV has been unsuccessful. Several latency biomarkers have been reported in the literature, but each of them defines only a fraction of the entire reservoir. The proposed candidate HIV latency biomarkers are CD2 [2], Fc fragment of IgG receptor IIa (*FCGR2A*, also known as CD32a) [3,4], lymphocyte activating 3 (*LAG3*), T cell immunoreceptor with Ig and ITIM domains (*TIGIT*), programmed cell death 1 (PD-1) [5], CEA cell adhesion molecule 1 (*CEACAM1*), and plexin B2 (*PLXNB2*) [6]. Each of these biomarkers facilitated up to 10-fold enrichment for latently infected cells from persons with HIV but could not capture the entire latent reservoir. These results were consistent with the idea that different latently infected cells likely express different sets of markers. Thus, to define all the cellular subsets of the latent HIV reservoir, a much more complex molecular panel is likely needed. Indeed, a more successful biomarker panel identified by the cytometry by time of flight (CyTOF) analysis, was comprised of eight molecules, both up- and downregulated [7].

The outstanding question that remains is what cellular and viral factors contribute to the variation of markers on different latently infected cells. Several mechanisms of the latent reservoir establishment have been proposed, including infection of activated, dividing cells that subsequently return to quiescence [8,9,10], and direct infection of resting cells [11,12,13,14]. Remarkably, the latent reservoir was established preferentially in minimally activated, non-dividing CD4+ T cells [15]. While evidence suggests that in resting CD4+ T cells, HIV provirus is more likely to be inactive [16], other studies demonstrated that depending on the environment, productive HIV infection is possible in these resting cells [17,18]. The strength of T cell receptor (TCR) signaling at the time of infection affects characteristics of the latent reservoir, such as the ability to reactivate with various latency reversing agents [19]. We hypothesize that such reservoirs may differ by their molecular signatures. Consistent with this idea, the biomarker panel proposed by Neidleman and colleagues was able to enrich for cells with inducible provirus from people with HIV [7].

To better understand how early responses to HIV infection contribute to the variation in signatures of the latent reservoir later in time, the present study aimed to comprehensively characterize transcriptomic remodeling induced by HIV infection in CD4+ T cells with variable proliferative responses to the TCR stimulus. To conduct gene expression profiling, we took the advantage of the single-cell RNA sequencing (scRNA-Seq) technology developed by 10X Genomics, Inc. (Pleasanton, CA, USA). This platform allows for sequencing of up to 10,000 cells in one experiment and uses antibodies against cell surface proteins to hash cells based on their desired characteristics. The desired characteristic in our experiment was the number of cell divisions undergone by cells following TCR stimulation: cells that responded well to the TCR stimulus and proliferated the most (referred to as “cells that divided many times”), cells that had a moderate proliferative response to the TCR stimulus (referred to as “cells that divided a few times”), and cells with no induced proliferation (referred to as “non-dividing cells”). Different cell subsets were hashed with antibodies designed to recognize the same proteins ubiquitously expressed on all lymphocytes (β2-microglobulin and β3 Na+/K+ ATPase CD298) but conjugated to different oligonucleotides (Biolegend, Inc., San Diego, CA, USA). A comparison of transcriptomic remodeling induced by HIV infection revealed both common and unique signatures for CD4+ T cell subsets with differential proliferative responses to the TCR stimulus. Some of these early gene expression changes were consistent with reported markers of latently infected cells, identified using in vitro models of HIV latency and cells from persons with HIV. Thus, it appears that certain molecular reprogramming in response to HIV infection is common for different stages of HIV infection, including long-term latency. The presence of transcriptomic changes that were unique for cells with different responses to the TCR stimulus is consistent with the idea that the latency biomarkers may depend on the cellular proliferative state at the time of infection.

## 2. Materials and Methods

### 2.1. Samples

Primary CD4+ T cells were isolated from the whole blood of three different HIV-seronegative donor volunteers and kept viably frozen until the beginning of the experiment. Following the thaw (day −2), cells were cultured overnight in RPMI medium containing 5% human serum, L-glutamate, penicillin, and streptomycin (RPMI) and then stained with a viable dye carboxyfluorescein diacetate succinimidyl ester (CFSE, day −1). The following day, cells were infected with wild-type HIV virus NL4-3 for 4–6 h and then stimulated in 6-well plates coated with αCD3/αCD28 antibodies (day 0). On day 4, cells were removed from plates and incubated for 3 additional days in RPMI medium that contained IL-2 and IL-15 to allow for propagation of the virus. On day 7, at the peak of viral infection as assessed using antibodies against Gag/p24 [15], cells were prepared for scRNA-Seq.

### 2.2. Preparation of Cells for scRNA-Seq and Sequencing

**Figure 1:**
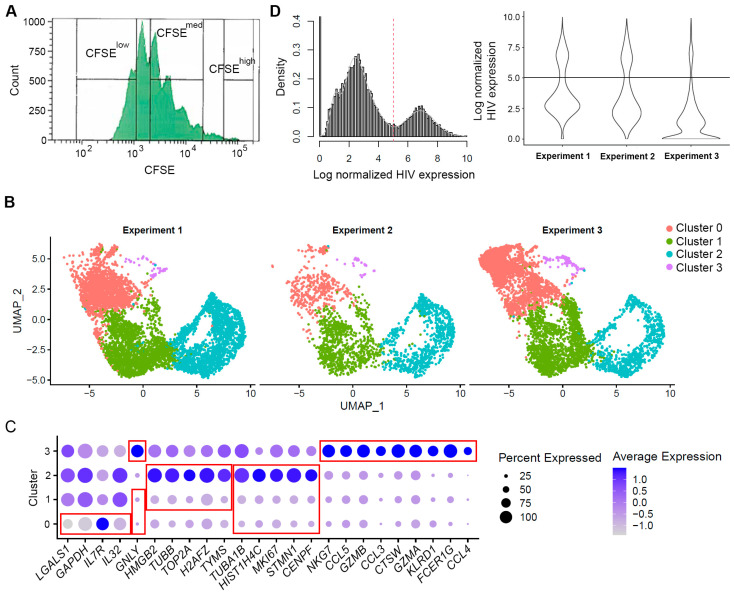
Cell sorting and phenotyping using scRNA-Seq data. (**A**) Gating strategy to sort cells that divided many times (CFSE^low^), few times (CFSE^med^), and non-dividing cells (CFSE^high^). For CFSE^low^ and CFSE^high^ populations, extreme cases were selected excluding cell populations in between. (**B**) Cell clustering following integration procedure demonstrating that phenotypically similar cells were present in each of the three experiments. Each dot represents an individual cell; X- and Y-axes represent the two-dimensional coordinates of each cell following the reduction of dimensionality of gene expression space using principal component analysis and uniform manifold approximation and projection (UMAP). (**C**) Cluster markers were identified using the *FindMarkers* function in the library *Seurat*, and the top 10 markers for each cluster (with the greatest absolute fold change in expression) were visualized using the DotPlot function. A number of genes were markers for more than one cluster (e.g., upregulated in one cluster while downregulated in another). Red boxes indicate gene markers for each of the clusters. The size of the circle indicates the percentage of cells where each marker is expressed; the color indicates the average level of expression (log normalized scaled UMI). (**D**) Visualization of HIV expression in cells. HIV expression exhibits bimodal distribution as shown for all cells in the integrated dataset (histogram on the left) and cells split by experiment (violin plot on the right). The red dotted line (left) and the black horizontal line (right) represent the identified threshold to separate the cells with high and low levels of HIV RNA.

Cells were stained with Aqua live/dead stain (Thermo Fisher, Inc., Waltham, MA, USA), and live cells were sorted using FACS Aria or the Sony MA900 Multi-Application cell sorter to separate live cells with robust (CFSE^low^), moderate (CFSE^med^), and no proliferative response (CFSE^high^) to the TCR stimulus (Figure 1A). CFSE^low^, CFSE^med^, and CFSE^high^ cells were incubated with cell hashing antibodies (Biolegend, Inc., San Diego, CA, USA) TotalSeq™-B0252, TotalSeq™-B0253, and TotalSeq™-B0254, respectively (please refer to Supplementary Methods for details). After staining, cells were mixed back together, 10,000 from each population, and 12,000 total cells were loaded into the Chromium Controller, aiming to achieve the targeted recovery of 10,000 cells in the scRNA-Seq experiment. Reverse transcription to generate cDNA and library preparation for scRNA-Seq were conducted per the manufacturer’s instructions (10X Genomics, Inc., Pleasanton, CA, USA) using v3 kits. Sequencing was conducted using the NovaSeq 6000 instrument (Illumina, Inc., San Diego, CA, USA) at the Institute of Genomics Medicine (IGM) Genomics Center, University of California San Diego. The data were provided in the form of paired .fastq files.

### 2.3. ScRNA-Seq Read Mapping and Counting

Raw sequencing .fastq files were used as input into the read mapping and counting software, CellRanger v4.0, developed by 10X Genomics, Inc. (Pleasanton, CA, USA). The Genome Reference Consortium Human Reference 38 combined with the HIV genome reference was used for mapping with default parameters for CellRanger that account for MAPQ adjustment, examination of compatibility with the transcriptome, unique molecular identifiers (UMI) counting, and cell-calling. The output filtered_feature_bc_matrix folder, which contains the barcodes after cell-calling filtration, was used for downstream analyses. The raw and mapped data are available through the Gene Expression Omnibus (GEO) database, accession number GSE223756.

### 2.4. ScRNA-Seq Data Pre-Processing

All the data were pre-processed in Bioconductor R (version 4.1.0), using libraries *Seurat* (version 4.0.3) for scRNA-Seq data and *ggplot2* (version 3.3.4) for graphs. First, the filtered_feature_bc_matrix data generated by the CellRanger were read in using the *Read10X* function in the library *Seurat*. To ensure that cell clustering occurs based on host gene expression and that differential gene analysis is not affected by HIV, HIV UMIs were removed from the gene expression matrix prior to creating the *Seurat* objects. HIV UMI, natural log-transformed HIV UMI, percent reads mapping to mitochondria genes, and UMI for all antibodies and their natural log-transformed values were added to the object metadata.

Next, we used a data-driven approach to exclude cells of poor quality (dead cells and multiplets) from the dataset. To determine filtering thresholds, either the interquartile range (IQR) or the Gaussian mixture model was used based on the data (see Supplementary Methods for details). Briefly, thresholds for filtering based on percent reads mapping to mitochondria genes were identified first. Next, thresholds for identifying cells positive for each of the three hashes—TotalSeq™-B0252, TotalSeq™-B0253, and TotalSeq™-B0254—were identified, and the information about the number of cell divisions based on these thresholds was added to the metadata. Cells with high percent reads mapping to mitochondria genes were removed, resulting in the removal of cells with a low number of detected genes (see Supplementary Methods). Next, cells representing true multiplets (positive for more than one hash) were removed from the objects.

### 2.5. Integrating Triplicate Experiments into One Dataset and Phenotypic Assessment of Cells

Since our experiment was conducted in triplicate, using cells from three different donors, we next integrated the data together using the anchoring procedure in the library *Seurat* [20]. Genes that were not expressed in any of the cells in any of the experiments were removed (23,171 genes were retained); data were normalized for library size and log-transformed. Two thousand genes with the most variation in expression were used to find integration anchors. Expression of these anchor genes was used to align phenotypically similar cells from the three experiments during the data integration process. Data were then scaled, and dimensionality reduction was first performed using principal component analysis (PCA), followed by finding neighbors for each cell (*FindNeighbors* function in the library *Seurat*) and identifying cell clusters (*FindClusters* function in the library *Seurat*). Our goal was to be able to distinguish the cells with differential responses to the TCR stimulus based on gene expression profiles; thus, we expected to identify only three major clusters, corresponding to the cells that divided many times, a few times, or remained non-dividing. Therefore, the resolution parameter in the *FindClusters* function was set to a relatively small value, 0.15. Following cluster identification, clusters were visualized via the implementation of the t-distributed stochastic neighborhood embedding (tSNE) or uniform manifold approximation and projection (UMAP) algorithms. This analysis verified that cells from the three experiments contained clusters of phenotypically similar cells (Figure 1B), suggesting the absence of much unexpected technical or biological variation between replicates. The markers of each cell cluster were then identified using the *FindMarker* function in the library *Seurat* to conduct unsupervised phenotypic characterization of these cells (Figure 1C and Table S1). Default parameters were used for this function, except min.pct was set to 0.25. The integrated dataset was used for all analyses going forward.

### 2.6. Identification of Genes Modulated by HIV Infection

HIV expression in individual cells was normalized to the total library size for that cell before the assessment of HIV expression levels in cells using the Gaussian mixture model described above and in the Supplementary Methods. Three distinct cell populations were identified: cells with no detectable HIV expression (HIV UMI = 0), cells with low HIV expression, and cells with high HIV expression, which formed two distinct peaks in the data (Figure 1D). Differential expression analysis between groups (e.g., HIV-negative cells vs. cells with high levels of HIV RNA) was conducted using the *FindMarkers* function in the library *Seurat*; the default parameters were used. These analyses were conducted independently for CFSE^low^ (hashed with TotalSeq™-B0252), CFSE^med^ (hashed with TotalSeq™-B0253), and CFSE^high^ (hashed with TotalSeq™-B0254) cells. The identified differentially expressed genes were compared between cell subsets. To ensure that the differences between the biomarkers identified for different cell subsets (i.e., cell division status) were not due to random chance, the permutation technique was used to randomly permute cell division status labels 100 times. Differential gene expression analysis between HIV-negative cells and cells with high levels of HIV RNA was conducted on each of the permuted datasets to construct the null distribution; empirical *p*-values were calculated using this null distribution (see Supplementary Methods for details).

### 2.7. Identification of Gene Ontology (GO) Terms and Pathways Over-Represented for Differentially Expressed Genes

We employed the over-representation-based enrichment analysis using a one-sided Fisher’s exact test (using hypergeometric distribution) and the pathways included in Molecular Signatures Database (MSigDB) v 7.5, Kyoto Encyclopedia of Genes and Genomes (KEGG), Reactome, and Gene Ontology (GO) databases using the R library *msigdbr* [21].

## 3. Results

### 3.1. Cell Filtering and Phenotypic Assessement

Despite taking extra care to ensure that cells in samples designated for scRNA-Seq are viable, some cells may become apoptotic during the sort for viable cells or shortly after the sort. These cells can be detected in scRNA-Seq data as those that have a high percentage of reads mapping to mitochondrial genes, or cells with a small number of genes detected. Filtering out cells of poor quality is an important step to facilitate data analysis.

In three replicate experiments, conducted using cells from three different blood donors, scRNA-Seq data were generated for 8715, 2913, and 6174 cells, respectively, for a total of 17,802 cells in all three experiments. Table 1 shows the summary of the read coverage in each experiment.

**Figure 2:**
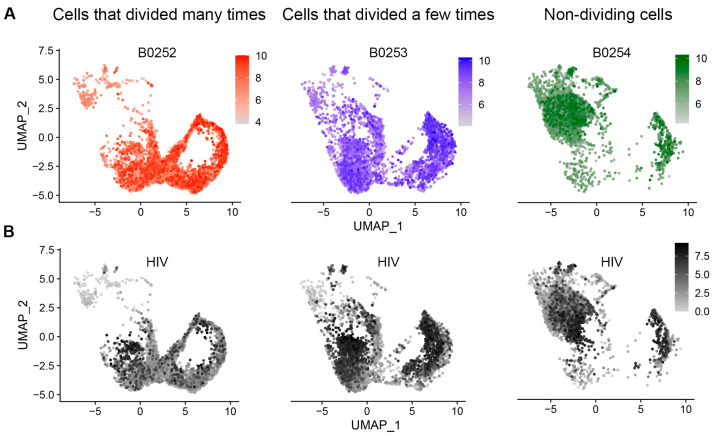
Characterization of cells by cell division status and the levels of HIV RNA. The cells that divided many or few times, or cells that remained non-dividing, were extracted from the integrated Seurat object. (**A**) Log-transformed UMIs of each of the antibody hashes were pasted on top of cell clusters produced by the unsupervised clustering based on cell transcriptomes (Figure 1B). (**B**) Log-transformed HIV UMIs were plotted for each cell subset.

Following the removal of cells with a high percentage of reads mapping to the mitochondria genes and having a small number of genes detected, the final datasets contained 6436, 2139, and 5334 cells, respectively. To reduce the number of tests during differential gene expression analysis, non-informative genes (not expressed in any of the cells) were removed, resulting in a total of 23,171 genes available for further analyses. Following the integration of the three datasets, the final set contained 13,909 cells, of which 4264 cells divided many times, 4136 cells divided a few times, and 4047 were non-dividing cells (Figure 2A). The remaining 1462 cells did not have any hashes. We first conducted the analysis of cell phenotypes based on unsupervised clustering (Figure 1B). The four identified clusters had distinct markers. Clusters 0 and 1 were characterized by the downregulation of genes involved in cell proliferation, while cells in cluster 2 had elevated expression of genes involved in cell proliferation (Figure 1C). Markers with the highest levels of expression in cluster 3 were markers of the effector cell subset with cytotoxic properties [22] (Figure 1C and Table S1). Clusters 0 and 3 overlapped with non-dividing cells (hashed with TotalSeq™-B0254), while clusters 1 and 2 were enriched for cells that divided a few or many times (hashed with TotalSeq™-B0253 and TotalSeq™-B0252, respectively) (Figure 1B vs. Figure 2A).

### 3.2. Evaluation of HIV Expression Levels in Different Cell Subsets

HIV expression levels (UMI) exhibited a bimodal distribution (Figure 1D). We used the Gaussian mixture model to identify a threshold to distinguish the two populations of cells, with high and low HIV RNA expression (threshold = 5.030465 log HIV UMI) (Figure 1D). Table 2 presents cell counts by the level of HIV expression and the number of cell divisions.

Cells with high expression of HIV RNA represented around 22% of all cells in the experiment. This number is comparable to the percentages of cells with p24 expression on day 7 post-infection, detectable by flow cytometry [15]. It is, therefore, possible that cells with high levels of HIV RNA expression observed in this dataset are the ones that actively produce infectious particles. The cells with low levels of HIV RNA may represent cells with low levels of protein production not detectable by flow cytometry or cells with transcriptionally active but translationally inactive proviruses. It is also possible that cells with particularly low HIV UMI (UMI = 1 or 2) represent infected cells in which integration has not yet occurred.

The majority of cells that expressed HIV RNA at high levels was found in the population of cells that divided a few times (Table 3 and Figure 2B). This observation is consistent with the idea that cells with moderate proliferative response to the TCR stimulus best support productive HIV infection. In non-dividing cells, HIV provirus might be less active. Cells that divided many times with active HIV infection might be more prone to death, while HIV provirus in the surviving cells might be less active.

### 3.3. Identification of Genes Modulated by Productive HIV Infection in Different CD4+ T Cell Subsets

**Figure 3:**
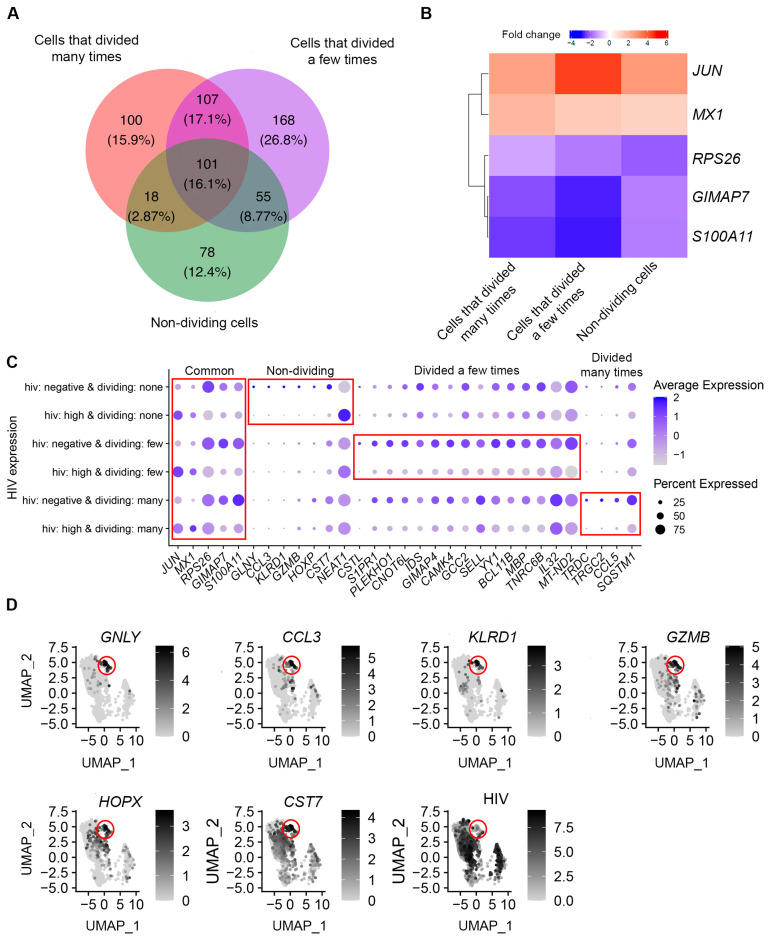
Identification of genes modulated by productive HIV infection in CD4+ T cell subsets with differential proliferative responses to the TCR stimulus. (**A**) A Venn diagram comparing genes differentially expressed between HIV-negative cells and cells with high levels of HIV RNA for cell subsets that divided many times, a few times, or remained non-dividing. (**B**) A heatmap of fold changes in gene expression modulation by HIV for genes that were identified as differentially expressed for each of the cell subsets, with absolute differences between the subsets greater than 1.5-fold. (**C**) Expression of genes that were identified as differentially expressed in common or uniquely for different cell subsets was visualized using the *DotPlot* function. The size of the circle indicates the percentage of cells where each marker is expressed; the color indicates the average level of expression (log normalized scaled UMI). The red boxes emphasize cases where genes were significantly differentially expressed between HIV-negative cells and cells with high levels of HIV RNA (Bonferroni-corrected *p*-value less than 0.05). (**D**) Non-dividing cells were extracted from the Seurat object. Log-transformed UMIs of selected genes differentially expressed between HIV-negative cells and cells with high levels of HIV RNA were plotted for the subset of non-dividing cells according to their position in phenotypic clusters (Figure 1B). Expression of these genes was enriched in cells from cluster 3 (red circle), which phenotypically represent cytotoxic effector cells. Cells infected with HIV were dys-enriched in this cluster (red circle). Each dot represents an individual cell; X- and Y-axes represent the two-dimensional coordinates of each cell following the reduction of dimensionality of gene expression space using principal component analysis and uniform manifold approximation and projection (UMAP).

Because HIV RNA expression exhibited bimodal distribution, and the percentage of cells with high levels of HIV RNA coincided with previously observed proportions of cells expressing the p24 protein, we decided to characterize transcriptomic remodeling in cells with high and low levels of HIV RNA separately. We first compared gene expression in cells with high levels of HIV RNA (that is, greater than the identified threshold) to that in HIV-negative cells separately for each of the cell subsets with varia- ble proliferative responses to the TCR stimulus. For cells that divided many times, 326 differentially expressed genes were identifed; for cells that divided a few times—431 genes were identified; and for non-dividing cells—252 genes were identified (Table S2). One hundred and one genes (16.1%) were differentially expressed in common (Figure 3A).

Despite some commonalities in transcriptomic remodeling induced by HIV in different cell subsets, the degree of modulation of some genes by HIV varied. To focus on more biologically relevant differences, we thresholded any difference in modulation to 1.5 to identify five such genes: Jun proto-oncogene, AP-1 transcription factor subunit (*JUN*), MX dynamin like GTPase 1 (*MX1*), ribosomal protein S26 (*RPS26*), GTPase, IMAP family member 7 (*GIMAP7*), and S100 calcium-binding protein A11 (*S100A11*) (Figure 3B). *JUN* was upregulated, and *GIMAP7* and *S100A11* were downregulated the most in cells that divided a few times; *MX1* was upregulated the most in cells that divided many times; and *RPS26* was downregulated the most in non-dividing cells (Figure 3B,C).

Further, some genes were found to be differentially expressed only in one of the cell subsets: cells that divided many times, cells that divided a few times, or non-dividing cells. To test whether these differences were not due to random chance, we have randomly permuted (reassigned) cell division status for each cell and repeated the analysis 100 times (please see Supplementary Methods for details). This analysis demonstrated that the identified differences in transcriptomic remodeling between cell subsets were not random. To focus on stronger biological differences, we selected genes with absolute fold change in expression of greater than 1.5 between cells with high levels of HIV RNA expression and HIV-negative cells. With this additional criterion, 7 genes were uniquely modulated in non-dividing cells; 15 genes were uniquely modulated in cells that divided a few times; and 4 genes were uniquely modulated in cells that divided many times (Figure 3C). The highest negative fold changes (greater than 2- and up to 12.5-fold) were observed for genes identified for the non-dividing cell subset (Table S2 and Figure 3C). Interestingly, when the expression of these genes was visualized on unsupervised cell clusters, it was evident that they were highly expressed on non-dividing cells phenotypically similar to cytotoxic effector cells (Figure 3D). This cluster was also dys-enriched for cells infected with HIV. Therefore, the most likely explanation for these results is the reduced susceptibility of non-dividing cytotoxic effector cells to HIV infection compared to other non-dividing cells. To test this hypothesis, we conducted the Pearson’s Chi-squared test for count data (*chisq.test* function in R) for cytotoxic (cluster 3) vs. non-cytotoxic non-dividing cells. This analysis demonstrated statistically significant dys-enrichment (*p* < 0.001) for cells with high levels of HIV RNA among the non-dividing cells from the cytotoxic cell cluster (3.8%, compared to 21.4% among non-cytotoxic cells), and enrichment of HIV-negative cells (30.6%, compared to 9.8% among non-cytotoxic cells). The proportions of cells with low levels of HIV RNA were similar (66% in cytotoxic cells vs. 69% in non-cytotoxic cells, *p* = 0.45). These results are consistent with the idea that the cytotoxic non-dividing cells may indeed be less susceptible to HIV infection, and in particular, do not support productive HIV infection to the same level as the non-cytotoxic cells.

### 3.4. Functional Analysis of Genes Modulated by HIV during Productive Infection

**Figure 4:**
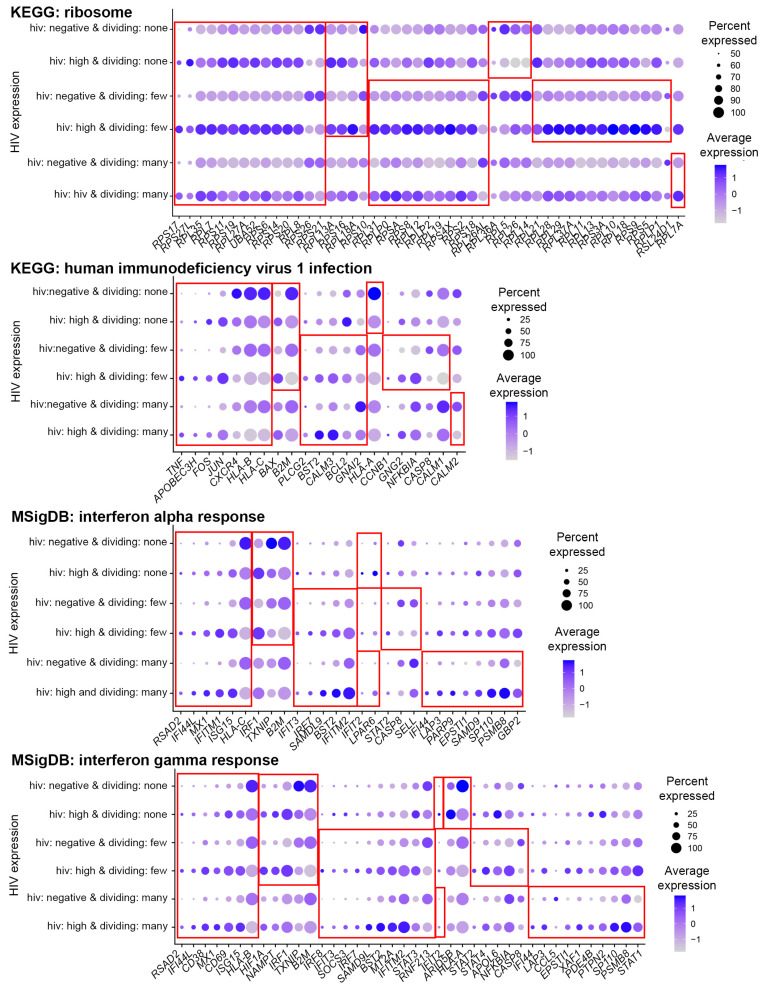
Expression of genes from pathways common for different CD4+ T cell subsets. Gene expression from each common pathway was visualized using the DotPlot function. All genes found at least for one cell subset were included. The size of the circle indicates the percentage of cells where each marker is expressed; the color indicates the average level of expression (log-normalized scaled UMI). The red squares emphasize cases when genes were significantly differentially expressed between HIV-negative cells and cells with high levels of HIV RNA (Bonferroni-corrected *p*-value less than 0.05).

To better understand the biology of response to HIV infection, we next conducted GO term and pathway gene set enrichment analyses for differentially expressed genes identified for each cell subset. Eleven GO terms were enriched for differentially expressed genes in common, with many terms related to ribosome and translation (Table S3). Thirty-five Reactome pathways were enriched for differentially expressed genes in common, with terms related to translation highly represented in this category. Among KEGG and MSigDB pathways, a lot of terms were found in common that were related to viral infection and interferon response (Table S3). These data indicate that regardless of the strength of proliferative responses to the TCR stimulus, HIV infection induces similar transcriptomic remodeling, common to infection with other viruses, such as influenza, cytomegalovirus, and SARS-CoV2 (see terms related to these infections in Table S3). Interestingly, even though the same processes were enriched for differentially expressed genes, specific genes involved and/or the magnitude of change of their expression in some cases was cell subset-dependent (Figure 4).

In addition to common responses, some biological processes were enriched for differentially expressed genes uniquely for each of the cell subsets: cells that divided many times, cells that divided a few times, or non-dividing cells. To focus on stronger biological differences, we considered terms that were found enriched in more than one category. For example, estrogen signaling terms were enriched for cells that divided many times in both KEGG (*estrogen signaling pathway*) and Reactome (*ESR-mediated signaling*) databases (Table S3). For cells that divided a few times, cellular senescence pathways were enriched in both KEGG and Reactome databases. Terms related to the cell division process were found both among GO terms (*spindle and spindle pole*) and in the MSigDB database (*G2M checkpoint*) (Table S3). Non-dividing cells shared terms related to immune responses in KEGG (*antigen processing and presentation*) and Reactome (*antigen presentation: folding, assembly,* and *peptide loading of class I MHC*) databases (Table S3).

**Figure 5:**
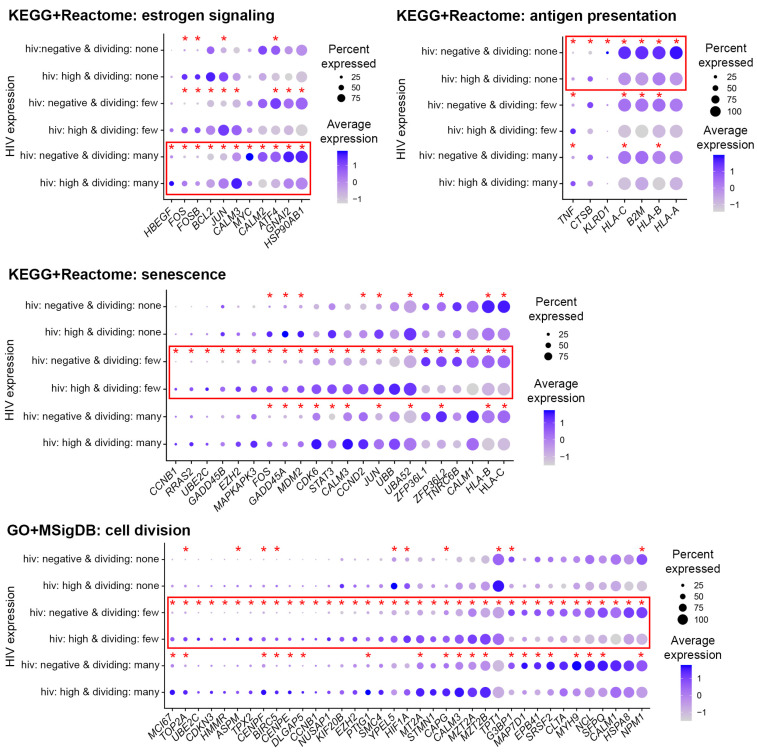
Expression of genes from pathways unique for different CD4+ T cell subsets. Genes from pathways related to the same process found in different databases were combined, and expression was visualized using the *DotPlot* function. The size of the circle indicates the percentage of cells where each marker is expressed; the color indicates the average level of expression (log-normalized scaled UMI). The red squares emphasize subsets for which pathways were significantly enriched for differentially expressed genes. The asterisks indicate individual genes that were significantly differentially expressed; in the case of cell subsets where pathway enrichment was not achieved, only some but not all genes were expressed differentially.

To gain a better understanding of the nature of the observed unique responses, we viewed the expression of individual genes across cells from the different cell subsets (Figure 5). Despite a GO term or pathway being enriched for differentially expressed genes in the case of a single cell subset, some genes from this GO term or pathway might be significantly differentially expressed in all three cell subsets (Figure 5). In other cases, the difference in gene expression was significant in two out of three cell subsets. Yet for other genes, the difference in expression did not reach statistical significance for more than one cell subset. These observations explain why a pathway or a GO term might be identified for one, but not other cell subsets as enriched for differentially expressed genes. Although in the majority of cases genes were modulated in the same direction in all cell subsets, sometimes it was not the case. For example, cathepsin B (*CTSB*) was significantly upregulated in response to HIV infection in non-dividing cells but was downregulated in dividing cells (Figure 5, KEGG+Reactome: antigen presentation). Overall, these results are consistent with the idea that the identified pathways are indeed modulated in a unique manner based on cellular proliferative responses to the TCR stimulus.

### 3.5. Identification of Genes Differentially Expressed in Cells with Low Levels of HIV RNA in Different CD4+ T Cell Subsets

**Figure 6:**
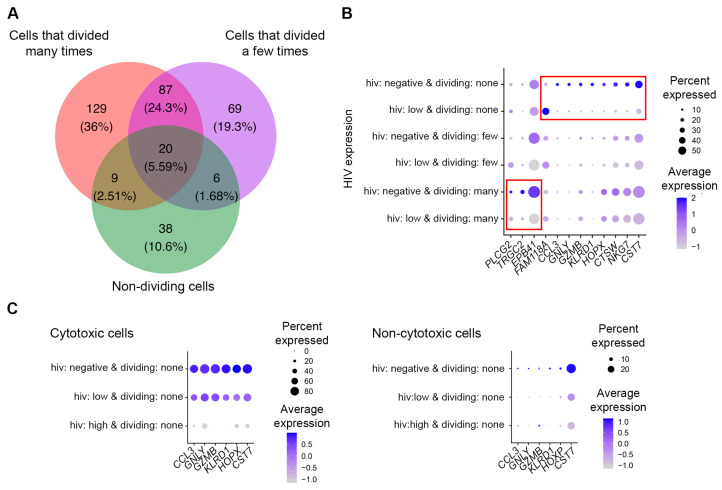
Identification of genes differentially expressed in cells with low levels of HIV RNA in different CD4+ T cell subsets. (**A**) A Venn diagram comparing genes expressed differentially between HIV-negative cells and cells with low levels of HIV RNA for cell subsets that divided many times, a few times, or remained non-dividing. (**B**) Expression of genes identified as differentially expressed uniquely for different cell subsets was visualized using the *DotPlot* function. The size of the circle indicates the percentage of cells where each marker is expressed; the color indicates the average level of expression (log-normalized scaled UMI). The red boxes emphasize cases where genes were significantly differentially expressed between HIV-negative cells and cells with low levels of HIV RNA (Bonferroni-corrected *p*-value less than 0.05). (**C**) Expression of cytotoxic genes in non-dividing cells from the cytotoxic cell cluster (cluster 3 in Figure 1) and non-cytotoxic cells. The general trend is the downregulation of the cytotoxic genes by HIV. The size of the circle indicates the percentage of cells where each marker is expressed; the color indicates the average level of expression (log-normalized scaled UMI).

Next, transcriptome remodeling was assessed in cells with low levels of HIV RNA expression: 245 differentially expressed genes were identified for cells that divided many times, 182 genes for cells that divided a few times, and 73 genes for non-dividing cells (Table S4). Twenty genes (5.6%) were modulated in common (Figure 6A). Similar to the observations with productively infected cells, genes modulated by HIV infection uniquely in cells with differential proliferative responses to the TCR stimulus were also present (Table S4). When genes were filtered based on the fold change in expression (absolute fold change greater than 1.5), three genes were uniquely modulated in non-dividing cells and eight genes were uniquely modulated in cells that divided many times (Figure 6B). The majority of these genes (except family with sequence similarity 8 member A, *FAM118A*) were downregulated in response to HIV infection. Six genes with the highest negative fold changes overlapped with the gene set identified for productively infected cells: C-C motif chemokine ligand 3 (*CCL3*), granulysin (*GNLY*), killer cell lectin like receptor D1 (*KLRD1*), granzyme B (*GZMB*), HOP homeobox (*HOXP*), and cystatin F (*CST7*), mapping to the cluster of cells with cytotoxic effector phenotype. Because the proportions of cells with low levels of HIV RNA were similar between non-dividing cells in the cytotoxic cell cluster and the rest of the non-dividing cells (see Section 3.3), this observation cannot be explained by the resistance of cytotoxic cells to low levels of HIV infection. We therefore accessed transcriptomic remodeling of these genes by HIV infection separately in cytotoxic and non-cytotoxic cells. As expected, only a small percentage of non-dividing HIV-negative cells found outside of the cytotoxic cell cluster expressed these genes, while expression of these genes was high in the majority of the cells in the cytotoxic cluster (Figure 6C). These genes were downregulated in the presence of HIV infection; the reduction of expression was the most pronounced in cytotoxic cells that expressed high levels of HIV RNA, although even low levels of HIV expression caused the reduction of expression of these genes in cytotoxic cells (Figure 6C). Overall, these results are consistent with the idea that even low levels of HIV infection induce the downregulation of cytotoxic genes, possibly reflecting the inhibition of antiviral responses by the incoming virus.

### 3.6. Functional Analysis of Genes Modulated by HIV in Cells with Low Levels of HIV RNA

The analysis described for productively infected cells was conducted using cells that had low levels of HIV RNA. Among categories enriched for differentially expressed genes in common for dividing and non-dividing cells, there were multiple GO terms and Reactome pathways related to ribosome and translation, and Reactome and KEGG pathways related to viral infection (Table S5). Even though overall fewer terms were enriched for genes modulated in cells with low levels of HIV RNA compared to cells with high levels of HIV RNA, a total of 39 GO terms and pathways (67.2%) were shared between the datasets. Seventeen (29.3%) were unique for cells with productive HIV infection, and two (3.4%) for cells with low levels of HIV RNA. These data indicate that regardless of the strength of proliferative responses to the TCR stimulus and the activity of the HIV provirus, HIV infection induces similar transcriptomic remodeling. The terms unique for productive infection included additional terms related to translation and viral infection. The unique terms identified for cells with low levels of HIV RNA were *endosomal/vacuolar pathway* and downregulation of *SMAD2/3:SMAD4 transcriptional activity* (both from the Reactome database).

In addition to common responses, some GO terms and pathways were uniquely enriched for differentially expressed genes for some, but not all cell subsets. Terms related to the cell division process prevailed among GO terms, KEGG, and Reactome pathways for cells that divided many or few times (Table S5). Pathways related to senescence were enriched for genes modulated in cells that divided a few times; pathways related to antigen processing and presentation were enriched for genes modulated in non-dividing cells (Table S5). As similar results were obtained with productively infected cells; it appears that some transcriptomic changes that vary across cell subsets with different proliferative responses to the TCR stimulus are independent of the activity of the HIV provirus. However, other responses appear to also depend on the HIV transcriptional activity (Table S5).

## 4. Discussion

ScRNA-Seq is a powerful method to conduct gene expression profiling in individual cells. Combined with the ability to hash cells using antibodies conjugated to different oligonucleotides enabled our study, which, for the first time, profiled gene expression in individual cells that differed by their proliferative responses to the TCR stimulus. Unsupervised transcriptomic analysis of CD4+ T cells in the present study identified four major clusters (Figure 1B), two of which were enriched for non-dividing cells, while the other two contained a mixture of cells that divided a few or many times (Figure 1C and Figure 2A). Thus, hashing the cells based on the number of cell divisions facilitated a better separation of these cells in our experiments, as opposed to solely relying on unsupervised phenotypic clustering following the exposure to the TCR stimulus.

The main objective of this study was to identify gene expression signatures associated with HIV infection in CD4+ T cell subsets with differential proliferative responses to the TCR stimulus. This objective was inspired by the ultimate goal of understanding how markers of latently infected cells are developed, and the fact that the strength of the TCR signaling at the time of infection affects the characteristics of the latent reservoir [19].

Multiple transcriptomic studies used either microarrays or RNA-Seq, and most recently scRNA-Seq aiming to identify signatures of active HIV infection [23,24,25,26,27,28,29,30,31,32,33]. While our study corroborated many of the results from these prior studies, it provided additional in-depth information regarding which cells, dividing or non-dividing, displayed certain transcriptomic responses to HIV infection. For example, interferon response was a common signature identified in multiple studies, including in vitro infection [23,32,33], in vivo responses of activated CD4+ T cells [25], and total CD4+ T cells obtained from viremic people with HIV [24,29,30,31]. Consistent with these observations, terms related to interferon signaling were enriched for differentially expressed genes in our study. However, our study design allowed us to determine that a number of interferon-stimulated genes were differentially modulated in non-dividing and dividing cells. For example, some of the HIV restriction factors induced by interferons, such as interferon-induced transmembrane protein 1 (*IFITM1*) and ISG15 ubiquitin-like modifier (*ISG15*), were induced by HIV infection in all cells; however, bone marrow stromal cell antigen 2 (*BST2*, also known as tetherin) and *IFITM2* were upregulated only in dividing cells, particularly cells that divided many times (Figure 4). Other genes, involved in interferon gamma response, AT-Rich Interaction Domain 5B (*ARID5B*), and major histocompatibility complex class I, A (*HLA-A*), exhibited unique modulation in non-dividing cells (Figure 4).

Responses to HIV infection specific to non-dividing and dividing cells were also observed in prior studies that were conducted without separating the cell subsets. For example, the *antigen processing and presentation* pathway was identified by Langer and colleagues [27], and *oxidative stress* by Shytaj et al. [28]. Our present study demonstrated that *antigen processing and presentation* was the most pronounced response in non-dividing cells (Figure 5 and Table S3), while the Reactome pathway *oxidative stress-induced senescence* was enriched for differentially expressed genes only for cells that divided a few times (Table S3). It is likely that when cells are experimentally activated to facilitate infection with HIV, such as was done in the Shytaj et al. study, signatures of dividing cells overwhelm any signal that comes from the non-dividing cells, which represent the minority. A signature of non-dividing cells (*antigen processing and presentation*) identified by Langer and colleagues [27] is likely explained by spinoculation being their method of cell infection. In our study, we were able to profile signatures of all cell subsets because sorted cells were added to each sequencing sample in equal proportions.

The identified differentially expressed genes in any transcriptomic profiling study may represent the result of certain cell types being preferentially infected by HIV, or a specific cellular response to HIV infection. ScRNA-Seq offers a unique opportunity to differentiate between these two scenarios. For example, we have determined that the cluster of non-dividing, cytotoxic effector cells was enriched for uninfected cells and dys-enriched for cells with high levels of HIV RNA (Figure 1B and Figure 2B). We observed that cytotoxic genes had a higher expression on HIV-negative non-dividing, but not dividing cells (Figure 3C). Thus, the apparent downregulation of these genes in non-dividing HIV-infected cells could, in part, be the result of the cytotoxic effector cells being less permissive to HIV infection. Interestingly, the proportions of cells with low levels of HIV RNA were similar between the cytotoxic and non-cytotoxic cells, suggesting that cytotoxic cells do not support productive HIV infection, rather than being less permissive to HIV infection in general. Assessment of cytotoxic gene expression separately in cytotoxic and the rest of the non-dividing cells demonstrated that HIV infection did result in downregulation of the cytotoxic genes (Figure 6C), which may be reflective of inhibition of antiviral responses by the incoming virus. Thus, the downregulation of cytotoxic genes by HIV infection in the present study represents both the result of the specificity of cells not supporting productive HIV infection and the cellular responses to HIV. The cytotoxic effector memory cell clones were enriched for HIV-positive cells in people with HIV during viremia and after viral suppression on antiretroviral therapy [34]. It is possible that due to low levels of HIV gene expression in cytotoxic cells, they survive and expand preferentially. Surprisingly, upregulation of cytotoxic genes, and in particular *GZMB* in people with HIV, was reported [34], which could be the result of the host immune response that developed over longer-term infection (20–48 days, compared to 7 days in the present study). The majority of other genes in the present study were modulated in cells within the major clusters of dividing and non-dividing cells; therefore, we conclude that, in most cases, they were modulated in response to HIV and did not reflect preferential support of HIV infection.

**Figure 7:**
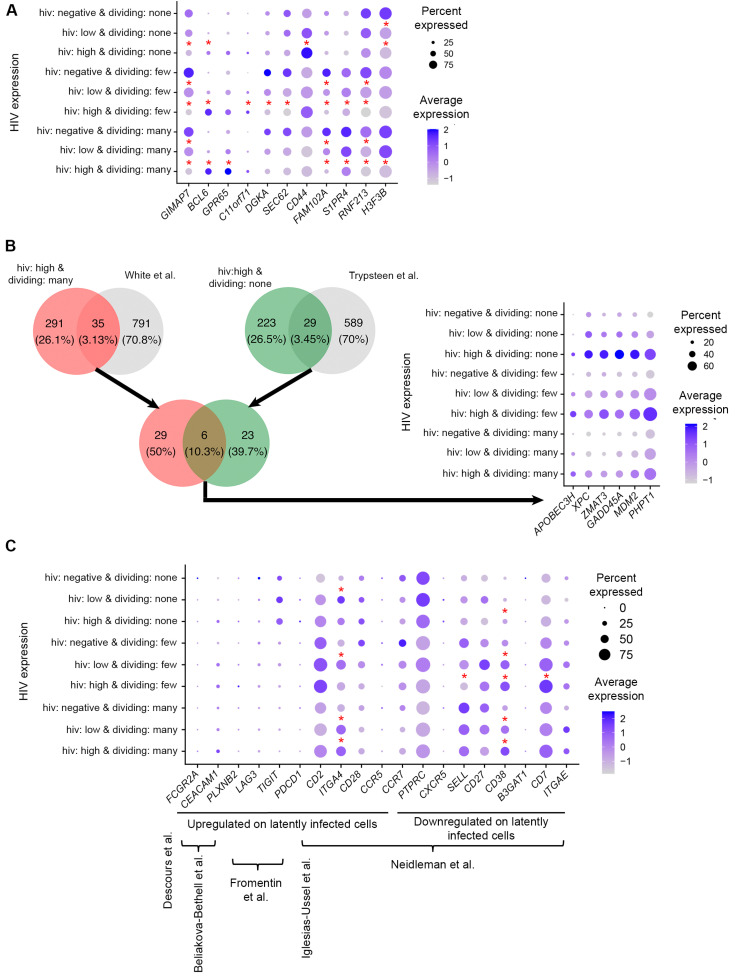
Evaluation of the identified differentially expressed genes as long-term markers of HIV infection, including latent infection. (**A**) Expression of genes modulated in the time course experiment conducted by Bauby et al., 2021 [32] was evaluated 7 h post-infection in our study using dot plots. (**B**) Differentially expressed genes identified in this study were compared to transcriptomic signatures of HIV latency reported previously in the literature. Genes modulated in cells that divided many times and had high levels of HIV RNA were compared to genes associated with latency established in vitro in productively infected cells that subsequently returned to quiescence [35]; genes modulated in non-dividing cells with high levels of HIV RNA were compared to genes modulated in latency established in vitro directly in resting cells [36]. The dot plot shows the expression of genes that were identified in common for these two comparisons. (**C**) Expression of latency biomarkers that were previously reported to enrich for latently infected cells from people with HIV was evaluated in different cell subsets from our study. A subset of markers associated with latency was shown to be modulated during initial HIV infection. In all dot plots, the size of the circle indicates the percentage of cells where each marker is expressed; the color indicates the average level of expression (log-normalized scaled UMI). Descours et al. [3]; Beliakova-Bethell et al. [6]; Fromentin et al. [5]; Iglesias-Ussel et al. [2]; Neidleman et al. [7]. Asterisks in (**A**,**C**) indicate significant modulation of genes in the present study (Bonferroni-corrected *p*-value less than 0.05); all genes used for comparison in (**B**) were significant.

The limitation of our study is the short-term culture. It cannot, therefore, be concluded, solely from our results, that the observed signatures during productive infection have relevance to HIV latency. It is not known how long a certain gene expression change may persist. The time course conducted by Bauby and colleagues indicates that some genes are modulated as early as 4 h post-infection, and a subset of these transcriptomic changes remain in effect for 48 h [32]. When we compared these data to results obtained from our study 7 days post-infection, we found that some of these transcriptomic changes remain. For example, *GIMAP7* was consistently down- and BCL6 transcription repressor (*BCL6*) upregulated from 4 h to 7 days post-infection ([32] and the present study), regardless of the cellular proliferative response to the TCR stimulus (Figure 7A). Upregulation of G protein-coupled receptor 65 (*GPR65*) persisted until day 7 post-infection, but only in the subset of cells that divided many times (Figure 7A). Other long-term cell subset-dependent signatures included upregulation of chromosome 11 open reading frame 71 (*C11ORF71*) and downregulation of diacylglycerol kinase alpha (*DGKA*) and SEC62 homolog preprotein translocation factor (*SEC62*) in cells that divided a few times (Figure 7A). *CD44* molecule was downregulated from 4 to 48 h in the Bauby et al. study [32], but was insignificantly downregulated only in cells that divided many times in our study (Figure 7A). In contrast, it was significantly upregulated in non-dividing cells (Figure 7A). This result could be missed in the Bauby et al. study, since they activated the cells prior to infection, with the majority of cells being dividing. Further, estrogen-induced osteoclastogenesis regulator 1 (*EEIG1*, previously known as *FAM102A*), sphingosine-1-phosphate receptor 4 (*S1PR4*), and ring finger protein 213 (*RNF213*) were downregulated up to 7 days post-infection solely in dividing cells, while H3.3 Histone B (*H3-3B*, also known as *H3F3B*) was downregulated in cells that divided many times and non-dividing cells ([32] and the present study, Figure 7A). A subset of these genes was also modulated in cells with low levels of HIV RNA (Figure 7A), consistent with the idea that some signatures are shared in cells with different proviral activity.

To further evaluate whether signatures of productive infection may be relevant to latency, we conducted the comparison of differentially expressed genes identified in the present study to genes that were found associated with in vitro latency established following productive infection [35] or in resting cells via cell-to-cell virus transmission from productively infected cells [36]. Thirty-five genes that were found differentially expressed in cells that divided many times and had high levels of HIV RNA were found to overlap with genes that were associated with latency established after productive infection [35] (Figure 7B). Twenty-nine genes that were found differentially expressed in non-dividing cells with a high level of HIV RNA were found to overlap with genes that were associated with latency established after infection of resting cells [36] (Figure 7B). These results are consistent with the idea that some markers of latently infected cells originate from the time when the virus in these cells was active. Furthermore, only six genes from these two lists were common: zinc finger matrin-type 3 (*ZMAT3*), growth arrest and DNA damage inducible alpha (*GADD45A*), MDM2 proto-oncogene (*MDM2*), alipoprotein B mRNA editing enzyme catalytic subunit 3H (*APOBEC3H*), phosphohistidine phosphatase 1 (*PHPT1*), and XPC complex subunit, DNA damage recognition and repair factor (*XPC*) (Figure 7B). Such a small overlap suggests that at least some of the transcriptomic responses to the initial HIV infection in dividing and resting cells are consistent with the signatures of HIV latency established in activated cells returning to quiescence and resting cells, respectively. Of note, we also detected unexpected overlaps in gene expression signatures between dividing cells in this study and latency established in resting cells [36] as well as non-dividing cells in this study and latency established in activated cells returning to quiescence [35]. These prior studies were conducted using the in vitro models of HIV latency comprised of mixtures of latently infected and uninfected cells, where the latter represent the majority. It is, therefore, not entirely possible to distinguish between signatures of latency and the effects of the exposure of the uninfected cells to the virus during culture. We suspect that some of these unexpected overlapping signatures may be the result of exposure of cells to the virus in these prior studies. The present study conducted at the single-cell level overcomes this limitation and allows for the profiling of transcriptomic signatures of cells that are infected.

Lastly, some of the latency biomarkers that enriched for latently infected cells from persons with HIV [2,3,5,6,7], were modulated on productively infected cells in our study (Figure 7C). Furthermore, some of these differences in gene expression were dependent on the strength of the cellular proliferative response to the TCR stimulus (Figure 7C). For example, integrin subunit alpha 4 (*ITGA4*, also known as CD49d) was upregulated in cells with low levels of HIV RNA in non-dividing cells and cells that divided a few times, and in all HIV-positive cells that divided many times. Selectin L (*SELL*, also known as CD62L) had a significantly lower expression and *CD7* had a significantly higher expression on cells with high levels of HIV RNA that divided a few times. *CD38* was upregulated in non-dividing cells with high levels of HIV RNA and all HIV-positive dividing cells (Figure 7C). *ITGA4*+*SELL*- markers were part of the panel developed by Neidleman et al. to enrich for latently infected cells [7]. Upregulation of *ITGA4* and downregulation of *SELL* in HIV-infected cells in this study are consistent with the idea that at least some of the initial responses to HIV infection may continue to persist into the latency stage long-term after infection. Thus, the identification of biomarkers using short-term in vitro systems and high-throughput sequencing with subsequent validation in individual latently infected cells from persons with HIV at the protein level is warranted as a viable approach to the identification of latency biomarkers.

## 5. Conclusions

In summary, our study demonstrated variable transcriptomic remodeling by HIV infection in cells with differential responses to the TCR stimulus. By further evaluating the existing literature on biomarkers of HIV latency, we conclude that some of the initial transcriptomic responses to infection may persist in cells for a long time and remain on latently infected cells. Despite the potential lack of specificity of a subset of latency markers, using these biomarkers for targeting the latent HIV reservoir would still be a viable strategy, with a caveat that productively infected cells will be also targeted.

The observed differential responses of dividing and non-dividing cells to initial HIV infection, which may continue into the latency stage, may explain, at least in part, the absence of a suitable biomarker, comprised of even more than a single molecule, that can define the entire cellular reservoir of the latent HIV provirus. The results from this study indicate the importance of considering different cell subsets that contribute to the latent reservoir when biomarkers of latency are identified. It is possible that other phenotypic differences in infected cells, such as the maturation stage, contribute to the biomarker heterogeneity. Further studies will be needed to dissect the complete molecular signatures of the different subsets of the latent HIV reservoir.

## Figures and Tables

**Table 1:** Summary of read coverage in the three replicate experiments.

	Experiment 1	Experiment 2	Experiment 3
Total number of cells	8715	2913	6174
Mean reads per cells	23,219	62,097	32,774
Median genes per cell	1689	2859	2050

**Table 2:** Cell counts by the level of HIV expression and the number of cell divisions.

Cell Subset	High HIV RNA	Low HIV RNA	HIV-Negative
Divided many times	406	3314	544
Divided a few times	1471	2351	314
Non-dividing	838	2779	430
Total	2715	8444	1288

**Table 3:** Percentages of cells with high, low, and no HIV RNA by cell division status.

Cell Subset	High HIV RNA	Low HIV RNA	HIV-Negative
% cells that divided many times	15.0	39.2	42.2
% cells that divided a few times	54.2	27.8	24.4
% non-dividing cells	30.9	32.9	33.4
Total	100	100	100

## Data Availability

ScRNA-Seq data were deposited to the Gene Expression Omnibus (GEO) database (URL: https://www.ncbi.nlm.nih.gov/geo) under accession number GSE223756.

## Supplementary Materials

The following supporting information can be downloaded at: https://www.mdpi.com/article/10.3390/pathogens12040511/s1, Supplementary Materials and Methods; Table S1: Cluster marker genes; Table S2: Genes expressed differentially between cells with high levels of HIV RNA and HIV-negative cells; Table S3: Gene ontology terms and pathways over-represented for the genes differentially expressed between cells with high levels of HIV RNA and HIV-negative cells; Table S4: Genes expressed differentially between cells with low levels of HIV RNA and HIV-negative cells; Table S5: Gene ontology terms and pathways over-represented for the genes differentially expressed between cells with low levels of HIV RNA and HIV-negative cells; Table S6: Feature barcoding and cell hashing antibodies; Table S7: Summary of all identified thresholds; Table S8: Identification of cells that represent doublets; Figure S1: Identification of thresholds to exclude cells stained with isotype antibody controls; Figure S2: Identification of thresholds to remove cells with high percentage of reads mapping to mitochondria genes; Figure S3: Filtering on cells with a high percentage of reads mapping to mitochondria genes results in the removal of cells with a low number of detected genes; Figure S4: Permutation results.
