# Supplementary material for: HIV Infection Elicits Differential Transcriptomic Remodeling in CD4+ T Cells with Variable Proliferative Responses to the T Cell Receptor Stimulus
Source: Pathogens. 2023 Mar 24;12(4):511. doi: 10.3390/pathogens12040511 (PMC10145558; doi:10.3390/pathogens12040511)
Supplement: Supplementary file 1 [file pathogens-12-00511-s001.zip › pathogens-2219971-Supplementary Methods.pdf]

Supplementary methods

Cell hashing

Antibodies used in these experiments are listed in **Table S6** below. In all experiments, CFSE^{low} population was hashed with TotalSeq™-B0252, CFSE^{med}, with TotalSeq™-B0253, and CFSE^{high}, with TotalSeq™-B0254. CFSE^{high} population of non-dividing cells was also stained with antibodies to differentiate cell maturation subsets (CD45RA and CD62L) and activation state (CD69), see **Table S6**. Following incubation with these antibodies, CFSE^{low}, CFSE^{med}, CFSE^{high} cells were pooled together in equal proportions, 10,000 per subset, and 12,000 cells of the pool were sampled for scRNA-Seq experiments. Additionally, in experiments 2 and 3, an aliquot of CFSE^{med} cells was hashed with TotalSeq™-B0255 antibody and stained with isotype controls, TotalSeq™-B0090 and TotalSeq™-B0092. Five hundred of these control cells were added to the sequencing samples. Maturation, activation, and isotype control staining were not used for this paper. Cell hashing antibodies were used at dilution of 1:8; all other antibodies were used undiluted.

Table S6. Feature barcoding and cell hashing antibodies.

Antibody	Specificity	Clone	Isotype	Barcode sequence	cat#
TotalSeq™-B0063	CD45RA	HI100	Mouse IgG2b, κ	TCAATCCTTCCGCTT	304161
TotalSeq™-B0147	CD62L	DREG-56	Mouse IgG1, κ	GTCCCTGCAACTTGA	304849
TotalSeq™-B0146	CD69	FN50	Mouse IgG1, κ	GTCTCTTGGCTTAAA	310949
TotalSeq™-B0090	Isotype control	MOPC-21	Mouse IgG1, κ	GCCGGACGACATTAA	400185
TotalSeq™-B0092	Isotype control	MPC-11	Mouse IgG2b, κ	ATATGTATCACGCGA	400379
TotalSeq™-B0252	Cell hash	LNH-94; 2M2	Mouse IgG1, κ	TGATGGCCTATTGGG	394633
TotalSeq™-B0253	Cell hash	LNH-94; 2M2	Mouse IgG1, κ	TTCCGCCTCTCTTTG	394635
TotalSeq™-B0254	Cell hash	LNH-94; 2M2	Mouse IgG1, κ	AGTAAGTTCAGCGTA	394637
TotalSeq™-B0255	Cell hash	LNH-94; 2M2	Mouse IgG1, κ	AAGTATCGTTTCGCA	394639

Data pre-processing

1.5 IQR rule. In statistics, the interquartile range (IQR) is a measure of statistical dispersion, defined as the difference between the 75th percentile (Q_3) and the 25th percentile (Q_1), i.e., $IQR = Q_3 - Q_1$. The interquartile range is a concept that is often used to find outliers in data in statistics. Data points that fall below $Q_1 - 1.5 * IQR$ or above $Q_3 + 1.5 * IQR$ are recognized as outliers. Our IQR function takes two parameters, the data to threshold and the IQR we wish to use.

***Mixture model rule*.** A mixture model is a statistical model for identifying the subpopulations in an overall population. It is one of the unsupervised learning methods, i.e., it does not require the subpopulation identity information. In statistical language, a mixture model models the distribution of observed measures to be a mixture distribution composed of the distributions of measures in the subpopulations. One of the most commonly used mixture models is a Gaussian mixture model, in which the overall population is a combination (mixture distribution) composed of more than one different Gaussian distribution (subpopulation). We used the Expectation-Maximization (EM) algorithm output for mixtures of normal distributions from R package *mixtools*. The identified threshold is the value of natural log-transformed UMI on which the probability of the cell coming from subpopulation on the left equals the probability of it coming from the right.

Our thresholding function has four parameters, including data to threshold (e.g. natural log-transformed UMI of hashes); the number of modes/peaks/subpopulations in the data (kkt); the vector of initial values for the mean locations of modes/peaks/subpopulations (muv.init); and the random seed. The parameters kkt and muv.init are determined visually from the data; however, in some cases several combinations of kkt and muv.init should be tried, in order to identify the best fit for the data. This happens when some of the modes/peaks/subpopulations are not very well defined; however, separate peaks are likely to be present (see examples below). We have selected these parameters based on the best model fit, indicated by the largest loglike value in the output of the threshold function. Finally, the number of clusters was selected by minimizing the Bayesian information criterion (BIC) statistic. The code is available at https://github.com/coralzhang/HIV_scRNA.

Identification of thresholds from actual data. To make all the datasets uniform, cells stained with TotalSeq™-B0255 were removed from experiments 2 and 3, which contained minor

populations of control cells stained with isotype antibodies. To do this, we first applied a log(x+1,e) transformation on the hash counts. Distribution of these transformed measures was generally bi(multi)-modal, indicating that there was more than one subpopulation in the overall population. Two major subpopulations were expected since cells exposed (positive) and not exposed (negative) to the antibody were mixed together. Thus, we used the mixture model to identify thresholds for TotalSeq™-B0255 (see **Figure S1** below).

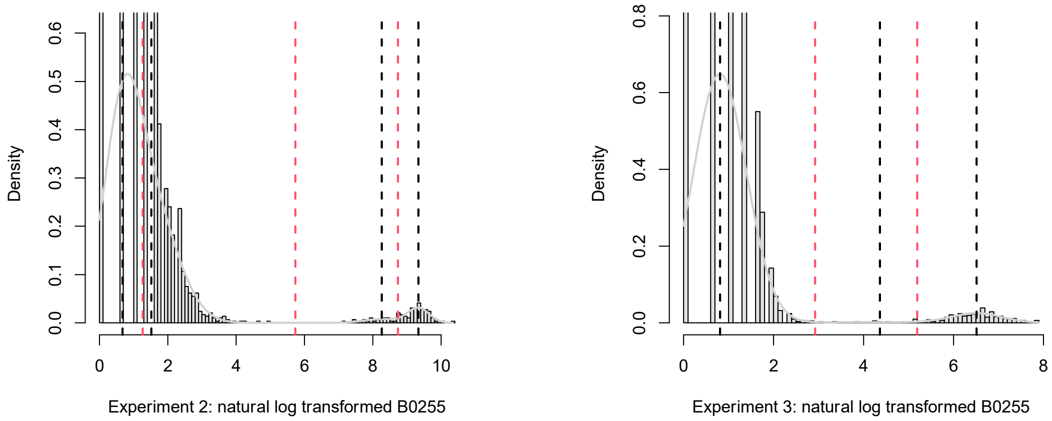

Figure S1. Identification of thresholds to exclude cells stained with isotype antibody controls. The grey curve represents the overall density estimation; the black dashed lines represent the centers of the subpopulations, and the red dash lines represent the thresholds estimated. *X axis*, the natural log-transformed value of B0255 UMI; *Y axis*, density, which represents the scaled frequency counts for cells in each bin so that the histogram has the total area of one. The histogram on the left illustrates experiment 2. Here the expected negative and positive populations are present. The positive population has two subpopulations/peaks at log B0255 UMI 8 and 9, and the negative population appears to show peaks at both 1 and slightly over 2; therefore, we tried both kkt=3 and 4 and chose kkt=4 based on its lower BIC statistics. The histogram on the right illustrates experiment 3, where eventually 3 peaks were chosen.

After filtering cells on identified thresholds, the next step was to remove dead or dying cells based on a high percentage of mitochondria reads. To do this, we first removed cells with a percentage of mitochondria reads higher than 40%. The distribution of this variable is generally unimodal and right-skewed, i.e., there is only a small number of cells with ultra-high percent of reads aligning to mitochondria genes. Given the skewed unimodal data, we used the IQR rule to define the thresholds (**Figure S2** below).

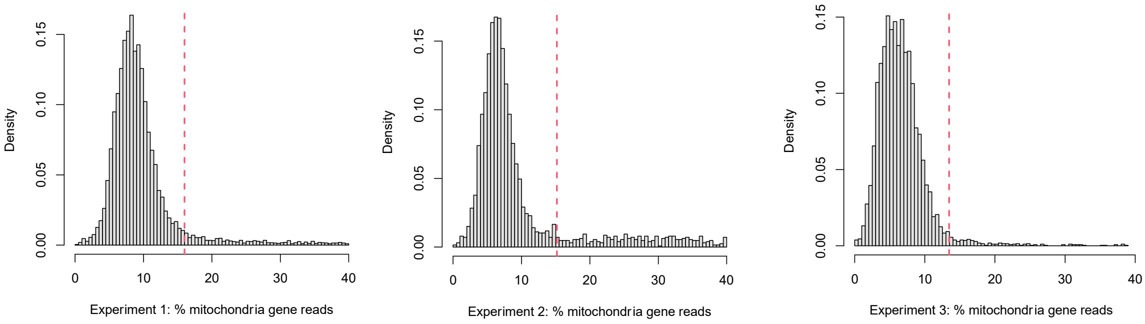

Figure S2. Identification of thresholds to remove cells with high percentages of reads mapping to mitochondria genes. The red dashed lines represent the identified thresholds. *X axis*, percent mitochondria gene reads in each experiment; *Y axis*, density, which represents the scaled frequency counts for cells in each bin so that the histogram has the total area of one.

One of the recommendations from the *Seurat* developers is filtering out cells with a low number of detected features (genes), as another measure of getting rid of bad-quality cells. We have assessed cell quality following the removal of cells with percentages of mitochondria reads above the identified thresholds. This analysis demonstrated that filtering data based on percentages of mitochondria reads removed essentially all cells with a low number of detected genes (**Figure S3**).

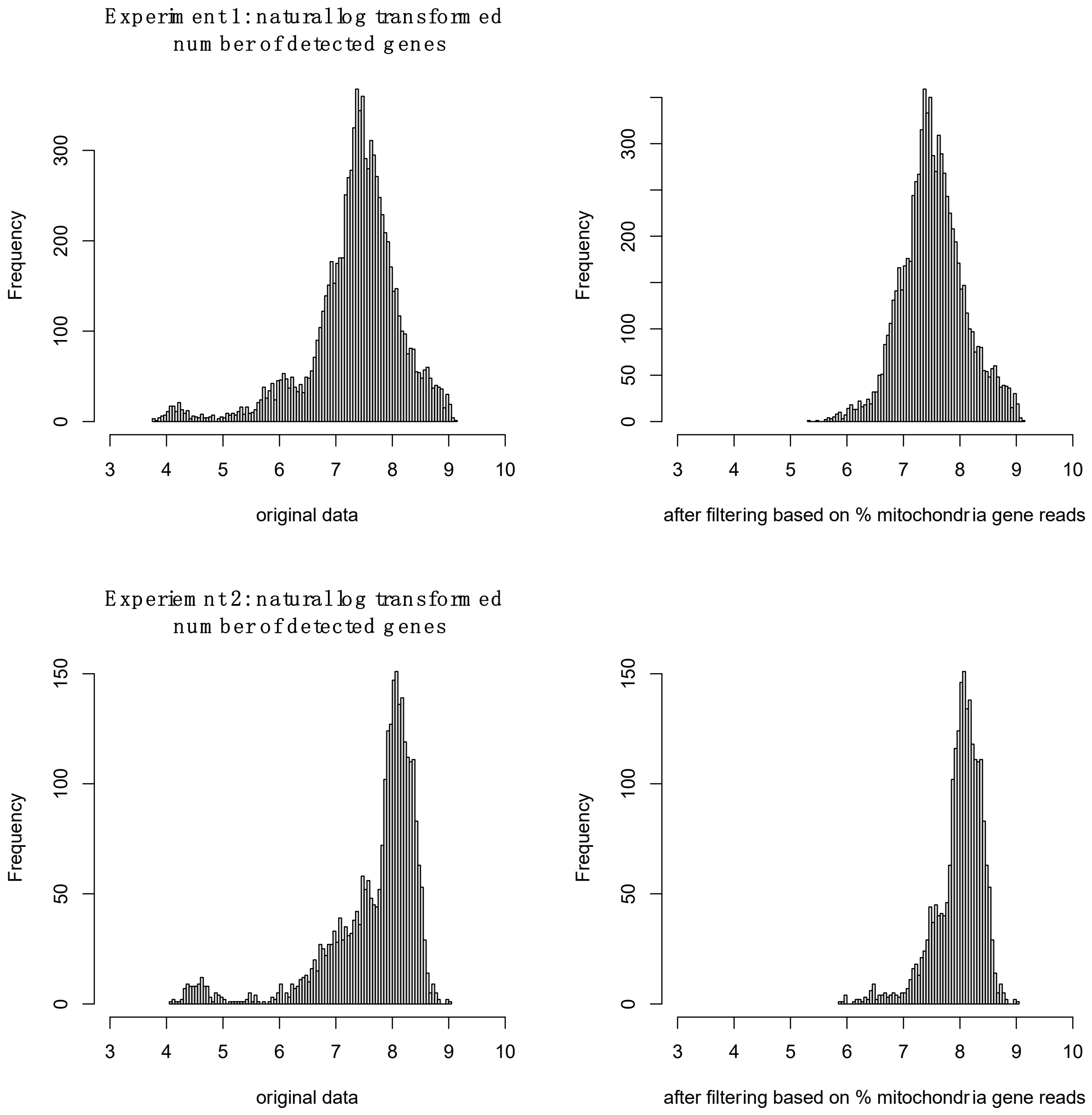

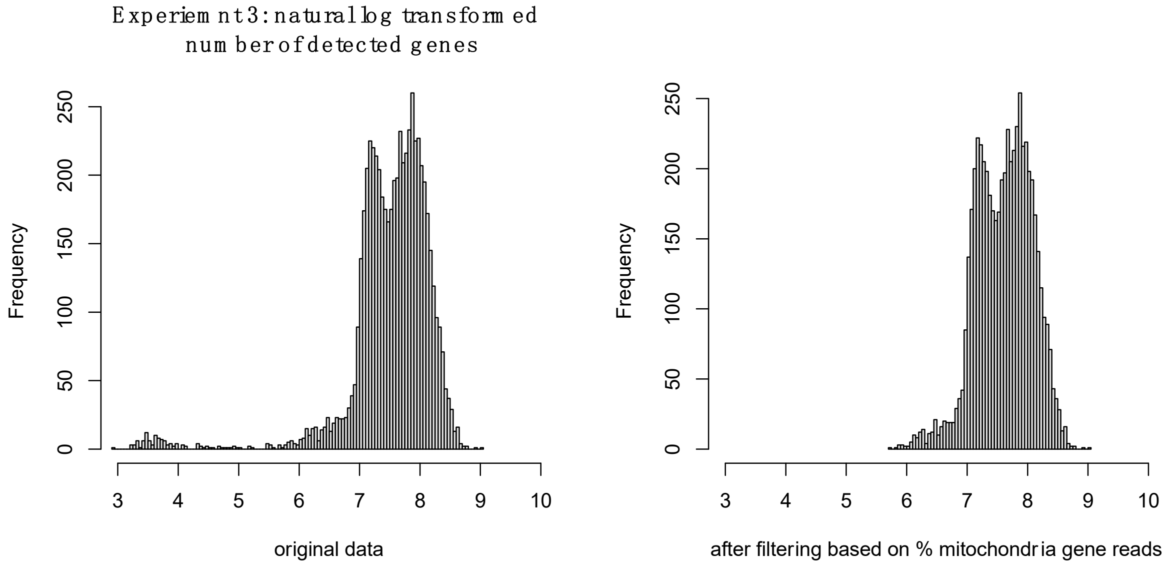

Figure S3. Filtering on cells with a high percentage of reads mapping to mitochondria genes results in the removal of cells with a low number of detected genes. Histograms depict cell distributions before (left) and after (right) filtering out cells based on a high percentage of reads mapped to mitochondria genes. *X axis*, natural log-transformed number of detected genes; *Y axis*, frequency, which represents the number of cells in each bin.

Next, we identified thresholds for each of the cell hashes, TotalSeq™-B0252, TotalSeq™-B0253, and TotalSeq™-B0254 in a similar manner as for TotalSeq™-B0255 described above. This information was further used to identify ground truth cell multiplets (droplets that contained more than one hash). **Table S7** below summarizes all thresholds identified during data pre-processing.

Table S7. Summary of all identified thresholds.

	B0255 (log scale)	% Mitochondria gene reads	B2052 (log scale)	B0253 (log scale)	B0254 (log scale)
Experiment 1	NA	15.986278	5.064453	4.528374	4.5834315
Experiment 2	5.731278	15.1820739	5.563767	5.039104	5.940909
Experiment 3	2.924681	13.462866	3.816704	3.990669	4.300492

We defined a cell as a doublet if two out of the three hashes (B0252, B0253, and B0254) were called positive by the thresholding method. The number and the percentages of doublets in each sample are summarized in **Table S8**.

Table S8. Identification of cells that represent doublets.

	Experiment 1	Experiment 2	Experiment 3
# of doublets	1281	59	270
Total # of cells after filtering B0255	8715	2813	5924
Percentage of doublets	14.7	2.1	4.6

Permutation technique to validate true differences in identified differentially expressed genes

After defining the cell division status (non-dividing cells, cells divided a few times, and cells divided many times), we first ran the *FindMarkers* function in the library *Seurat* on three cell populations to identify genes expressed differentially between HIV-negative and HIV-positive cells. For non-dividing cells and cells that divided many times, a total of 386 differentially expressed genes were identified, among which 106 (27.46%) were shared by non-dividing cells and cells that divided a few times. For cells divided a few times and cells divided many times, a total of 483 differentially expressed genes were identified, among which 180 (37.27%) were shared by cells that divided a few times and cells that divided many times. For cells divided a few times and non-dividing cells, a total of 469 differentially expressed genes were identified, among which 138 (29.42 %) were shared by cells that divided a few times and cells that divided many times. To investigate the significance of the overlap of the differentially expressed genes between cells with the different cell division statuses, we randomly permuted (reassigned) the cell division status for each cell, keeping the same population sizes (keeping the number of non-dividing cells, cells that divided a few times and cells that divided many times the same), and ran the *FindMarkers* function again on these randomly re-assigned cell populations. We repeated this process 100 times, and for each iteration, we recorded the number and percentage of differentially expressed genes shared by comparing different division levels, which constitute the null distributions. We then plotted a histogram of these values and the

actual observed values from the real data, demonstrating that the effect of cell division status was indeed significant (**Figure S4**, empirical *p*-value=0).

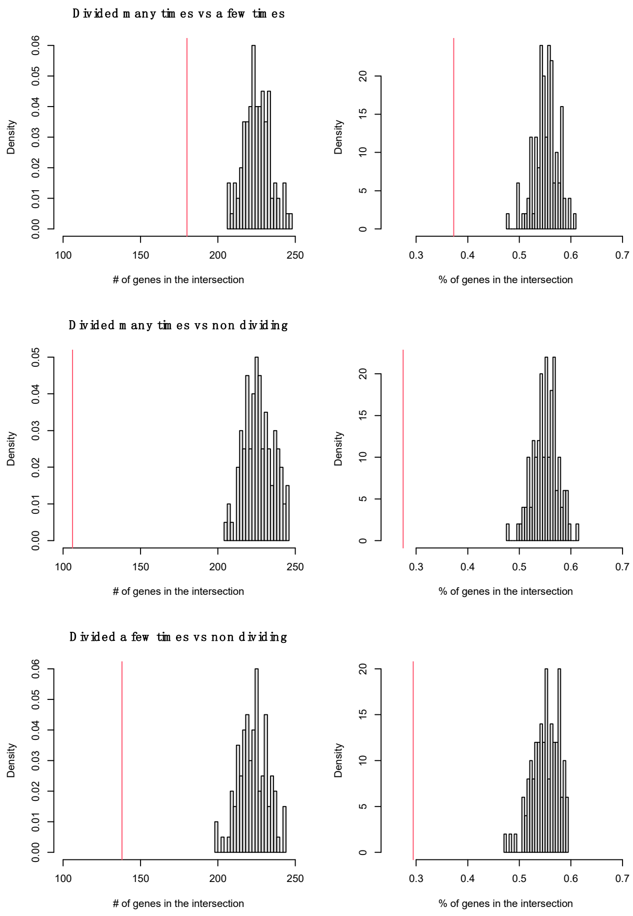

Figure S4. Permutation results. The histograms of the number (left) and percentage (right) of markers of the HIV-positive cells shared by cells that divided many times and cells that divided a few times (top), cells that divided many times *vs* non-dividing cells (middle) and cells that divided a few times *vs* non-dividing cells (bottom) in the 100 permutations. The red vertical lines represent the actual observed values in the real data.
